# Traditional complementary and alternative medicine (TCAM) use among PLHIV on antiretroviral medication

**DOI:** 10.1186/s12981-024-00673-w

**Published:** 2024-11-20

**Authors:** Mawulorm KI Denu, Maame Araba E. Buadu, Frederick Adrah, Cornelius A. Normeshie, Kofi Poku Berko

**Affiliations:** 1 Department of Medicine, University of Massachusetts Chan Medical School, Worcester, MA USA; 2 Department of Medicine, Massachusetts General Hospital, Boston, MA USA; 3 Joint School of Nanoscience and Nanoengineering, University of North Carolina, Greensboro, NC USA; 4 Department of Family Medicine, University of Virginia Medical School, Charlottesville, VA USA; 5 Department of Infectious Diseases, Korle-Bu Teaching Hospital, Accra, Ghana

**Keywords:** HIV/AIDS, Traditional, complementary and alternative medicine, Antiretroviral medications

## Abstract

**Background:**

Traditional complementary and alternative medicine (TCAM) are products and practices that differ from conventional allopathic medicine. There continues to be an increase in the use of these methods of treatment in developed and developing countries worldwide. This often owes to the perceived ability of these treatment methods to cure chronic medical conditions like HIV. However, TCAM use among PLHIV may be associated with reduced compliance with antiretroviral medications, resulting in poor viral load suppression and increased risk for opportunistic infections. The concomitant use of antiretroviral drugs and TCAM practices may be influenced by some sociodemographic and health-related factors.

**Objective:**

To determine the prevalence of TCAM use and examine the sociodemographic and health-related factors associated with its use among PLHIV on antiretroviral medications at the Infectious Disease unit of Korle-Bu Teaching Hospital in Ghana.

**Methods:**

A cross-sectional study was conducted among attendants at an adult HIV clinic. 420 study participants were selected by systematic sampling. Data related to TCAM use, sociodemographic and health-related factors were collected using a standardized questionnaire and patient chart review. Multivariate logistic regression model was used to determine the association between TCAM use, sociodemographic and health-related factors.

**Results:**

Of the 420 study participants, majority were female (76.2%) and urban community dwellers (77.9%). 77.4% of participants had been diagnosed with HIV for $\:\ge\:$ 5 years and had been on anti-retroviral medications for more than 5 years. The prevalence of TCAM use among PLHIV was 25.2%. No sociodemographic or HIV-related health factor was significantly associated with TCAM use in the study population.

**Conclusion:**

TCAM use was high among PLHIV. No sociodemographic or health-related factor was found to be associated with TCAM use. Further studies employing a qualitative approach using key informant interviews and focused group discussions are needed to explore reasons for its use. Care providers and policy-makers should look beyond sociodemographic and health-related factors in addressing TCAM use among PLHIV.

## Background

Traditional, complementary and alternative medicine (TCAM) refers to products and practices that differ from conventional medicine. They consist of biological-based products like plant herbs, faith-based or spiritual/religious healing practices and mind-body healing techniques such as acupuncture, yoga, and massage [1, 2]. TCAM use is widespread globally, with an estimated prevalence between 76% and 80% in the general population [3, 4]. The use of this nonconventional method of medicine continues to grow, especially across the African continent. This has led to the establishment of research centers and the introduction of curricula in universities in countries such as Ghana, Nigeria, and South Africa for the purpose of studying these methods [1]. This is similar to the trend noted in Europe and other Western countries [5, 6]. In Ghana, the use of TCAM among the general population is estimated to be between 70% and 86% [7, 8].

The practice of TCAM use is particularly common among individuals with chronic medical conditions [9]. The advent of antiretroviral medications has significantly reduced morbidity and mortality associated with Human Immunodeficiency Virus and Acquired Immune Deficiency Syndrome (HIV/AIDS), essentially rendering it a chronic medical condition [10].

The use of TCAM among persons living with HIV(PLHIV) is known to be relatively high, with a worldwide average prevalence of 50-95% [11]. Previous studies estimate the prevalence of TCAM use among persons living with HIV (PLHIV) in developed countries such as the USA to be about 60% [12], and about 53.2% in developing countries like Ghana [8]. The availability and affordability of TCAM, especially in poor rural communities of developing countries, have contributed to their high level of acceptance [13].

Age, sex, marital status, educational background, rural or urban residence, and other sociodemographic factors have been associated with TCAM use among PLHIV [9, 11, 14]. Some health-related factors such as duration of HIV diagnosis, duration of ART treatment and comorbid medical conditions have also been associated with its use. TCAM use is often associated with poor adherence to ART [10, 15]. Because the efficacy of antiretroviral treatment may be dependent on strict adherence, TCAM use may be problematic among PLHIV. An adherence rate of at least 95% is needed to maintain optimum viral load suppression and other positive treatment outcomes [16]. There has been concern about poor ART compliance with increase in adverse outcomes and drug resistance among PLHIV in communities with high levels of TCAM use.

With no previously published study on TCAM use among PLHIV from the southern parts of Ghana and at the Korle Bu Teaching Hospital, Ghana’s biggest teaching hospital, this study examined the level of TCAM use among attendants of an HIV at the Korle Bu Teaching Hospital in Ghana and explored the association between TCAM use and sociodemographic and health-related factors.

## Materials and methods

### Study design and data collection

This was a facility-based analytic cross-sectional study among attendants of the adult HIV clinic at Korle Bu Teaching Hospital between June and August 2020. The study was limited to PLHIV aged 18 years and above who had been on antiretroviral treatment for at least 6 months. A researcher-designed questionnaire was used for data collection on TCAM use, sociodemographic and health factors associated with its use among PLHIV that access care at the unit. Specific types of TCAM asked about were herbal mixtures and capsules, balms ointments and oils. A pilot study of 20 participants was conducted to validate and refine the questionnaire after initial design. In addition, participant health information such as duration of diagnosis, duration on antiretrovirals, HIV-typing, ART regimen, and viral load within the past year were extracted from health records and documented in the questionnaire.

Self-adherence to medications was ascertained for all participants using the Brief Adherence Rating Scale (BARS) and medication recall for 30 days. The study protocol (KBTH-IRB/00047/2020) was reviewed and approved by the Korle Bu Teaching Hospital Institutional Review Board on 27th May 2020. All enrolled participants provided a signed written consent and all the data collected were deidentified and kept confidential and accessible to approved study team members only.

### Dependent and explanatory variables

The dependent variable of interest was TCAM use. This was assessed in the survey by the response to the question, “Do you currently use traditional or alternative forms of medicines aside from the ones that your doctor prescribed?”

#### Sociodemographic factors

Data were collected on sociodemographic factors such as age, education, sex, religion, marital status, occupation, and region of residence using a standardized questionnaire. The region of residence was categorized into: “Rural,” “Peri-urban,” and “Urban.”

#### Health-related factors

Health-related factors examined in this study were HIV type, HIV viral load, duration of HIV diagnosis, duration of ART treatment, ART regimen, ART adherence rate, and comorbidities. These were obtained from patients using the questionnaire and review of patient health records.

ART adherence rate was assessed by a standardized 30-day recall of medications using the Brief adherence rating scale (BARS) and pill count. The low-cost, ease of administration, and minimal patient burden it offered made it favorable to carry out the study in an outpatient clinic [17].

### Statistical analyses

Descriptive statistics were first produced for sociodemographic and health-related variables. Following this, Chi-squared tests were performed to examine the differences in the independent variables between the two TCAM use categories of respondents. Multiple logistic regression was then utilized to examine the association between TCAM use and the predictor sociodemographic and health-related variables. All analyses were done with STATA (version 16). Statistical tests were 2-sided, and the significance level was set at 0.05.

## Results

### Sociodemographic characteristics of the Study Population

Table 1 summarizes the sociodemographic characteristics of respondents. Of the 420 participants enrolled in this study, 76.2% of respondents were female, and 82.1% were above 40 years old. The prevalence of TCAM use among study participants was 25.2%.

**Table 1:** Sociodemographic characteristics of study participants

Baseline Characteristics (*N* = 420)	*n* (%)
**Gender**	
Male	100 (23.8%)
Female	320 (76.2%)
**Age Category**	
< 40 years	75(17.9%)
$\:\ge\:$40 years	345(82.1%)
**Education**	
Primary	151 (35.9%)
Secondary	141 (33.6%)
Vocational/Technical	31 (7.4%)
Tertiary	32 (7.6%)
None	65 (15.5%)
**Residence**	
Rural	11 (2.6%)
Urban	327 (77.9%)
Peri-urban	82(19.5%)
**Employment Status**	
Formally employed	49 (11.7%)
Informally employed	323(76.9%)
Unemployed*	38(9.0%)
Retired	10(2.4%)
**TCAM use**	
Yes	106(25.2%)
No	314(74.8%)
**Type of TCAM used** ^**a**^	
Herbal supplements	89(84)
Non-herbal supplements	15(14.2)
Faith based (e.g. spiritual oils)	2(1.8)
**Comorbid medical conditions** ^**b**^	
Hypertension	74(67.3)
Diabetes	12(10.9)
Asthma	5(4.5)
Liver disease	3(2.7)
Others	16(14.6)

*Includes students ^**a−**^*n* = 106 ^**b−**^*n* = 110

### Sociodemographic factors and TCAM use

Table 2 summarizes the chi-square analysis results to examine the differences in sociodemographic factors between the two TCAM use categories. There was no significant difference in sociodemographic factors between respondents who used TCAM and those who did not.

**Table 2:** Chi-square analysis of sociodemographic factors by TCAM use category

Variables	TCAM use		*p*-value (χ^2^)
	Yes (*N* = 106)	No (*N* = 314)	
**Sex**			0.466
Male	28(26.4%)	72(22.9%)	
Female	78(73.6%)	242(77.1%)	
**Age Category**			**0.525**
< 40 years	17(16%)	58(18.5%)	
$\:\ge\:$40 years	89(84%)	256(81.5%)	
**Religion**			**0.215**
Christian	91(85.8%)	280(89.2%)	
Muslim	14(13.2%)	26(8.3%)	
Other	1(1%)	8(2.5%)	**`**
**Marital Status**			**0.585**
Single	23(21.7%)	70(22.2%)	
Married	41(38.7%)	131(41.7%)	
Widow/Widower	21(19.8%)	55(17.5%)	
Separated	3(2.8%)	18(5.7%)	
Divorced	8(7.5%)	23(7.3%)	
Cohabiting	10(9.5%)	17(5.6%)	
**Residence**			**0.885**
Rural	3(2.8%)	8(2.5%)	
Peri-urban	19(17.9%)	63(20.1%)	
Urban	84(79.3%)	243(77.4%)	
**Education level**			**0.856**
None	15(14.2%)	50(15.9%)	
Primary	38(35.8%)	113(36%)	
Secondary	38(35.8%)	103(32.8%)	
Vocational/Technical	9(8.5%)	22(7%)	
Tertiary	6(5.7%)	26(8.3%)	
**Employment Status**			**0.881**
Unemployed	9(8.5%)	29(9.2%)	
Formally employed	10(9.4%)	39(12.4%)	
Informally employed	84(79.2%)	239(76.1%)	
Retired	3(2.9%)	7(2.3%)	

### Health-related factors and TCAM use

Table 3 summarizes the results of the chi-square analysis to examine the differences in health-related factors between the two TCAM use categories. Again, there was no significant difference between the two TCAM use categories.

**Table 3:** Chi-square analysis of health-related factors by TCAM use category

Variables	TCAM use		*p*-value (χ^2^)
	Yes (*N* = 106)	No (*N* = 314)	
**HIV type**			**0.985**
Type 1	103(97.2%)	305(97.1%)	
Type 2	3(2.8%)	9(2.9%)	
**Duration of HIV diagnosis**			**0.769**
< 5 years	25(23.6%)	70(22.2%)	
5–10 years	40(37.7%)	110(35%)	
> 10 years	41(38.7%)	134(42.8%)	
**Duration on ART**			**0.906**
< 5 years	25(23.6%)	70(22.2%)	
5–10 years	38(35.8%)	109(34.7%)	
> 10 years	43(40.6%)	135(43.1%)	
**HIV viral load**			**0.154**
$\:\le\:$1000cp/ml	97(91.5%)	299(95.2%)	
> 1000cp/ml	9(8.5%)	15(4.8%)	
**Comorbidity**			**0.653**
Yes	26(24.5%)	84(26.8%)	
No	80(75.5%)	230(73.2%)	
**ART adherence rate**			**0.355**
$\:\le\:$95%	29(27.4%)	101(32.2%)	
> 95%	77(72.6%)	213(67.8%)	
**ART Regimen**			**0.886**
NNRTI-based	83(78.3%)	238(75.8%)	
INSTI-based	17(16%)	57(18.2%)	
PI-based	6(5.7%)	19(6%)	

### Adjusted multivariate logistic regression for the association between TCAM use and sociodemographic, health-related factors

The results of the logistic regression are summarized in Table 4. No sociodemographic and health-related factor was found to be significantly associated with TCAM use.

**Table 4:** Multivariate Logistic Regression for the association between TCAM use and sociodemographic, health-related factors

Variable	aOR (95%CI)	SE	*p*-value
Sex Female Male	*Ref* 1.35(0.75,2.44)	0.41	0.322
Age Category < 40 years $\:\ge\:$ 40 years	*Ref* 1.42(0.72,2.81)	0.41	0.322
**Religion** Christian Muslim Others	*Ref* 1.84(0.86,3.90) 0.33(0.04,2.81)	0.71 0.36	0.114 0.309
**Marital Status** Married Single Widow/Widower Separated Divorced Cohabiting	*Ref* 1.30(0.69,2.45) 1.31(0.67,2.56) 0.62(0.17,2.26) 1.25(0.50,3.14) 2.49(0.98,6.32)	0.42 0.45 0.41 0.59 1.18	0.417 0.424 0.466 0.634 0.054
**Residence** Rural Peri-urban Urban	*Ref* 0.72(0.16,3.19) 0.72(0.18,2.98)	0.55 0.52	0.666 0.654
**Education** None Primary Secondary Vocational/Technical Tertiary	*Ref* 1.23(0.60,2.55) 1.48(0.71,3.09) 1.69(0.58,4.89) 1.17(0.31,4.48)	0.46 0.56 0.92 0.80	0.569 0.301 0.334 0.818
**Occupation** Formally employed Informally employed Unemployed Retired	*Ref* 1.50(0.56,4.01) 1.29(0.39,4.26) 1.82(0.36,9.15)	0.75 0.79 1.50	0.420 0.672 0.465
**HIV Type** Type 1 Type 2	*Ref* 0.78(0.16,3.90)	0.64	0.764
**Duration of HIV diagnosis** < 5 years 5–10 years > 10 years	*Ref* 5.69(0.40,79.99) 0.88(0.47,1.67)	7.67 0.29	0.198 0.704
**Duration on ART** < 5 years 5–10 years > 10 years	*Ref* 0.17(0.01,2.29) 1.00	0.22 -	0.180 -
**HIV viral load** $\:\le\:$ 1000 cp/ml > 1000 cp/ml	*Ref* 1.41(0.40,4.91)	0.90	0.595
**Comorbid Condition** No Yes	*Ref* 0.90(0.52,1.56)	0.25	0.711
**ART adherence rate** $\:\le\:$ 95% > 95%	*Ref* 1.26(0.75,2.12)	0.33	0.383
**ART regimen** NNRTI-based INSTI-based PI-based	*Ref* 0.82(0.43,1.57) 0.97(0.32,2.89)	0.27 0.54	0.551 0.952

## Discussion

The results of this study indicate that approximately one out of every four persons who seek HIV care at the Infectious Diseases unit uses some form of TCAM. Although moderately high, this rate is lower than that observed in a similar study conducted at Komfo Anokye Teaching Hospital in the Ashanti region of Ghana, which reported the use of TCAM in 50% of clinic attendants [8]. The difference noted in rate between these two studies from different regions of the same country could be attributed to the difference in availability of TCAM and the number of rural communities located close to the centers where studies were conducted. The Ashanti region of Ghana has more rural communities where TCAM is more readily available for use, at a cheaper price. The prevalence rate from our study is also lower than the reported rates from similar studies from the Kwazu-Natal region of South Africa (51.3%) [18], Western Uganda (63.5%) [19], and Ethiopia(43.7%) [2]. Other literature in sub-Saharan Africa reported an average prevalence rate of about 45.8% [1]. However, the results were comparable to UK studies [4]. Most study respondents were from urban and peri-urban communities, where access to TCAM may be reduced, accounting for the relatively lower prevalence noted in our study. This prevalence, however remains considerably high, considering that all participants are HIV clinic attendants who have been on ARVs for at least 6 months, and have been previously counseled on ARV compliance and avoidance of TCAM use before starting ARV treatment. Commonly used TCAM types included herbal mixtures(66%), balms and oils(25%). These were used mainly to treat acute medical illnesses such as febrile illnesses, musculoskeletal conditions and common cold. Other study participants used them as an adjunct to manage chronic comorbid medical conditions like hypertension and diabetes, while some used TCAM as a way of boosting their immune systems. The sources of information regarding accessing TCAM was through word of mouth from other patients, television advertisement, radio and print media, which are assessable to all patients of all socio-demographic backgrounds.

Demographic factors have long been associated with TCAM use among PLHIV [1, 19, 20]. For example, the use of TCAM among PLHIV in some was previously reported to be associated with the female gender [1, 20]. Other studies have also shown an association between age and TCAM use among PLHIV [19]. The increased risk of comorbid conditions with age has been thought of as the main reason for this association [19]. However, neither gender nor age was found to be significantly associated with TCAM use in this study. Furthermore, other sociodemographic factors such as educational level, rural or urban settlement, and type of occupation were not found to be significantly associated with TCAM use in this study, although these were reported to be associated factors in a few studies in developed and developing countries [1].

Our results also show that comorbid disease conditions were not associated with TCAM use. This is a surprising finding as TCAM use was expected to be high among participants with chronic comorbid medical conditions seeking to cure or alleviate their symptoms with the use of TCAM. Hypertension was the most prevalent comorbid medical condition among study participants. The prevalence of hypertension in our study population was 21.2%, comparable to hypertension prevalence of 27% reported among PLHIV from studies in Nigeria and South Africa [21] and among the general population in Ghana of about 28% [22]. Although TCAM use among patients with hypertension in the general population is also well noted in other studies [23], a subgroup analysis of the comorbid disease conditions showed no association between hypertension and TCAM use in this study.

The use of TCAM in newly-diagnosed people with HIV was reported to be high, especially in the pre-ART era worldwide [24]. The impact of antiretroviral medications in improving treatment outcomes for PLHIV led to a gradual move away from the use of TCAM to treat HIV/AIDS. Some studies suggest that patients living with HIV for extended periods have higher odds of TCAM use due to the onset of comorbid medical conditions like diabetes and dyslipidemia [25]. The duration since diagnosis was therefore considered a predictor of TCAM use among PLHIV [24, 26]. In this study however, we found no association between duration since diagnosis and TCAM use. We note, however, that all our study participants have been diagnosed for durations greater than 6 months.

Optimum adherence to antiretroviral medications is objectively assessed by patient viral load. The results show no association between TCAM use and viral load. This suggests that the study participants who use TCAM are also highly adherent to ART treatment. The adherence rates calculated for the study participants confirm a high level of ART adherence. A high proportion of the study participants demonstrated an adherence rate of at least 95% and greater. There was no statistically significant association between the level of adherence and TCAM use. This showed that even though most patients adhered to ARV treatment, some still used TCAM. This highlights the fact that PLHIV are not using TCAM primarily to manage or cure HIV, but for other ailments. This may be due to the relative high cost of orthodox medications compared to their counterpart TCAM. The public health policy program in Ghana for HIV/AIDS care makes antiretrovirals free at all point of care facilities. Hence cost of antiretrovirals are not a limitation to care as compared to medications for other ailments such as hypertension and diabetes. The high level of adherence at this HIV care clinic can be attributed to the level of health care staff and clinic health promotion and educative practices on adherence to ART. PLHIV who attend rural clinics were found to have a nine-fold likelihood of using alternative medicines compared to those that attended urban clinics, like the Korle Bu Fevers unit [27]. The high compliance to ARV treatment and significant TCAM use among PLHIV further raises concerns of increased drug interactions and increased adverse effects in the event of any drug interactions between ARV treatment regimen and TCAM.

This study was conducted among patients who are regular attendants of an HIV clinic in an urban teaching hospital with high levels of ART adherence. They are regularly seen and educated on avoidance of TCAM and strict ART adherence by various well-trained staff, including clinical psychologists, public health nurses, medical officers, and specialists in HIV. This may explain the lack of association between the sociodemographic factors and TCAM use seen in some previous studies. While there was no association observed between TCAM use and the sociodemographic and health-related variables we examined, the level of TCAM use among study participants is still a concern. There is a need to explore TCAM use further by healthcare providers and policymakers, especially with the ready availability of ARTs. New insights using a qualitative research approach are necessary to examine emerging factors that may drive TCAM use, especially among ART-compliant patients. Healthcare providers and policy makers must approach interventions to addressing TCAM use among PLHIV holistically, and examine other factors beyond socio-demographic and health-related factors in designing their interventions. This will help design more-targeted and effective programs or strategies aimed at reducing TCAM use while on ARV treatment.

### Limitations

This was a single-site study with a cross-sectional design. A cause-effect relationship could therefore not be drawn for the association between TCAM use and the sociodemographic and health-related factors. In addition, a high proportion of participants had been diagnosed with HIV > 5 years and had high levels of adherence to ART treatment. Therefore, the results may not indicate the level of TCAM use among newly diagnosed, non-attendant, or non-compliant persons living with HIV.

The interviews conducted during clinic hours in the presence of healthcare providers may affect respondents’ willingness to disclose TCAM use. There may have also been recall bias among study participants.

## Conclusion

This study’s findings indicated that there remains a continuing high level of TCAM use among PLHIV on antiretrovirals. However, with no sociodemographic or health-related factor found to be associated with TCAM use, further studies employing a qualitative approach using key informant interviews and focused group discussions are needed to explore reasons for its use. In addition, care providers and policy-makers should look beyond sociodemographic and health-related factors in addressing TCAM use among PLHIV.

## Data Availability

Data collected and analysed for this study are available from the authors on reasonable request.

## Abbreviations

TCAM: Traditional, Complementary and Alternate Medicine
PLHIV: Persons Living with HIV
AIDS: Acquired Immune Deficiency Syndrome
ARV: Anti Retroviral

## References

[1] James PB, Wardle J, Steel A, Adams J. Traditional, complementary and alternative medicine use in Sub-saharan Africa: a systematic review. BMJ Glob Health. 2018;3:895. 10.1136/bmjgh-2018-000895. 10.1136/bmjgh-2018-000895 PMC6231111 30483405

[2] Endale Gurmu A, Teni FS, Tadesse WT. Pattern of Traditional Medicine Utilization among HIV/AIDS patients on antiretroviral therapy at a University Hospital in Northwestern Ethiopia: a cross-sectional study. Evidence-Based Complement Altern Med. 2017;2017. 10.1155/2017/1724581. 10.1155/2017/1724581 PMC5380827 28421118

[3] Misawa J, Ichikawa R, Shibuya A, Maeda Y, Arai I, Hishiki T, Kondo Y. The impact of uncertainty in society on the use of traditional, complementary and alternative medicine: a comparative study on visits to alternative/traditional/folk health care practitioners. BMC Complement Altern Med. 2019;19(1):251. 10.1186/s12906-019-2662-x. 31500604 10.1186/s12906-019-2662-x PMC6734350

[4] Mbutho NP, Gqaleni N, Korporaal CM. Traditional complementary and alternative medicine: knowledge, attitudes and practices of health care workers in HIV and AIDS clinics in Durban hospitals. Afr J Tradit Complement Altern Med. 2012;9(SUPPL3). 10.4314/ajtcam.v9i3S.8. 10.4314/ajtcam.v9i3s.8 PMC3746618 23983356

[5] Fjær EL, Landet ER, McNamara CL, Eikemo TA. The use of complementary and alternative medicine (CAM) in Europe. BMC Complement Med Ther. 2020;20(1):108. 10.1186/s12906-020-02903-w. PMID: 32252735; PMCID: PMC7137515. 32252735 10.1186/s12906-020-02903-w PMC7137515

[6] Nahin RL, Rhee A, Stussman B. Use of Complementary Health approaches overall and for Pain Management by US adults. JAMA. 2024;331(7):613–5. 10.1001/jama.2023.26775. 38270938 10.1001/jama.2023.26775 PMC10811586

[7] Kretchy IA, Boadu JA, Kretchy JP, et al. Utilization of complementary and alternative medicine for the prevention of COVID-19 infection in Ghana: a national cross-sectional online survey. Prev Med Rep. 2021;24:101633. 10.1016/j.pmedr. 2021.101633. Epub 2021 Nov 9. PMID: 34777985; PMCID: PMC8575551. 34777985 10.1016/j.pmedr.2021.101633 PMC8575551

[8] Kenu A, Kenu E, Bandoh DA, et al. Factors that promote and sustain the use of traditional, complementary and integrative medicine services at LEKMA hospital, Ghana, 2017: an observational study. BMC Complement Med Ther. 2021;21:14. 10.1186/s12906-020-03185-y. 33407386 10.1186/s12906-020-03185-y PMC7788857

[9] Abou-Rizk J, Alameddine M, Naja F. (2016) ‘Prevalence and Characteristics of CAM Use among People Living with HIV and AIDS in Lebanon: Implications for Patient Care.‘, *Evidence-based complementary and alternative medicine: eCAM*. Hindawi Limited, 2016, p. 5013132. 10.1155/2016/5013132 10.1155/2016/5013132 PMC5168459 28050191

[10] Bukenya D, Mayanja BN, Nakamanya S, Muhumuza R, Seeley J. What causes non-adherence among some individuals on long term antiretroviral therapy? Experiences of individuals with poor viral suppression in Uganda. AIDS Res Therapy. 2019;16(1):2. 10.1186/s12981-018-0214-y. 10.1186/s12981-018-0214-y PMC6340167 30665440

[11] Limsatchapanich S, Sillabutra J, Nicharojana LO. Factors related to the use of complementary and alternative medicine among people living with HIV/AIDS in Bangkok, Thailand. Health Sci J. 2013;7(4):436–46.

[12] Bahall M. Prevalence, patterns, and perceived value of complementary and alternative medicine among HIV patients: a descriptive study. BMC Complement Altern Med. 2017;17(1):422. 10.1186/s12906-017-1928-4. 28830419 10.1186/s12906-017-1928-4 PMC5567497

[13] Nnebue CC, et al. Herbal Medicine use in pregnancy among Nigerian women attending clinics in a Tertiary Hospital in Imo State. Am J Med Stud. 2016;4(1):1–10. 10.12691/ajms-4-1-1.

[14] Conboy L et al. (2005) Sociodemographic Determinants of the Utilization of Specific Types of Complementary and Alternative Medicine: An Analysis Based on a Nationally Representative Survey Sample, *The Journal Of Alternative And Complementary Medicine*. Available at: www.liebertpub.com (Accessed: 26 January 2020). 10.1089/acm.2005.11.977 16398589

[15] Sangeda RZ, et al. Predictors of non-adherence to antiretroviral therapy at an urban HIV care and treatment center in Tanzania. Drug, healthcare and patient safety. Volume 10. Dove; 2018. pp. 79–88. 10.2147/DHPS.S143178. 10.2147/DHPS.S143178 PMC6109655 30174460

[16] Adejumo OA, et al. Contemporary issues on the epidemiology and antiretroviral adherence of HIV-infected adolescents in sub-saharan Africa: a narrative review. Journal of the International AIDS Society. Volume 18. Wiley-Blackwell; 2015. p. 20049. 110.7448/IAS.18.1.20049. 10.7448/IAS.18.1.20049 PMC4575412 26385853

[17] Stirratt MJ, et al. Self-report measures of medication adherence behavior: recommendations on optimal use. Translational behavioral medicine. New York LLC: Springer; 2015. pp. 470–82. 10.1007/s13142-015-0315-2. 10.1007/s13142-015-0315-2 PMC4656225 26622919

[18] Peltzer K, Preez NF, Ramlagan S, Fomundam H. Use of traditional complementary and alternative medicine for HIV patients in KwaZulu-Natal, South Africa. BMC Public Health. 2008;8:255. 10.1186/1471-2458-8-255. Published 2008 Jul 24. 18652666 10.1186/1471-2458-8-255 PMC2503977

[19] Olisa NS, Oyelola FT. Evaluation of use of herbal medicines among ambulatory hypertensive patients attending a secondary health care facility in Nigeria. Int J Pharm Pract. 2009;17(2):101–5. 10.1211/ijpp.17.02.0005. 20214258

[20] Langlois-Klassen D, Kipp W, Jhangri GS, Rubaale T. Use of traditional herbal medicine by AIDS patients in Kabarole District, western Uganda. Am J Trop Med Hyg. 2007;77(4):757–63. 10.4269/ajtmh.2007.77.757. 17978084

[21] Nakaranurack C, Manosuthi W. Prevalence of Non-AIDS comorbidities and Factors Associated with metabolic complications among HIV-Infected patients at a Thai Referral Hospital. J Int Association Providers AIDS Care (JIAPAC). 2018b;17:232595741775225. 10.1177/2325957417752256. 10.1177/2325957417752256 PMC6748460 29357771

[22] Dosoo, D. K., Nyame, S., Enuameh, Y., Ayetey, H., Danwonno, H., Twumasi, M., … Asante,K. P. (2019). Prevalence of Hypertension in the Middle Belt of Ghana: A Community-Based Screening Study. *International Journal of Hypertension*, *2019*. https://doi.org/10.1155/2019/1089578. 10.1155/2019/1089578 PMC6800906 31687204

[23] Asfaw Erku D, Basazn Mekuria A. Prevalence and correlates of complementary and alternative Medicine Use among Hypertensive patients in Gondar Town, Ethiopia. Evidence-Based Complement Altern Med. 2016;2016. 10.1155/2016/6987636. 10.1155/2016/6987636 PMC5097805 27843480

[24] Nlooto M, Naidoo P. Traditional, complementary and alternative medicine use by HIV patients a decade after public sector antiretroviral therapy roll out in South Africa: a cross sectional study. BMC Complement Altern Med. 2016;16(1):128. 10.1186/s12906-016-1101-5. 27189225 10.1186/s12906-016-1101-5 PMC4869398

[25] Levy M, Greenberg A, Hart R, Powers Happ L, Hadigan C, Castel A. High burden of metabolic comorbidities in a citywide cohort of HIV outpatients: evolving health care needs of people aging with HIV in Washington DC. HIV Med. 2017;18(10):724–35. 10.1111/hiv.12516. 28503912 10.1111/hiv.12516 PMC5643214

[26] Ankrah DNA, Koster ES, Mantel-Teeuwisse AK, Arhinful DK, Agyepong IA, Lartey M. Facilitators and barriers to antiretroviral therapy adherence among adolescents in Ghana. Patient Prefer Adherence. 2016;10:329–37. 10.2147/PPA.S9669154. 27042024 10.2147/PPA.S96691 PMC4801129

[27] Laar AK, Kwara A, Nortey PA, Ankomah AK, Okyerefo MPK, Lartey MY. Use of non-prescription remedies by Ghanaian Human Immunodeficiency Virus- positive persons on antiretroviral therapy. Front Public Health. 2017;5:26. 10.3389/fpubh.2017.00115. 28603710 10.3389/fpubh.2017.00115 PMC5445137

